# Postoperative air leak grading is useful to predict prolonged air leak after pulmonary lobectomy

**DOI:** 10.1186/s13019-017-0568-6

**Published:** 2017-01-23

**Authors:** Sang Gi Oh, Yochun Jung, Sanghoon Jheon, Yunhee Choi, Ju Sik Yun, Kook Joo Na, Byoung Hee Ahn

**Affiliations:** 1 Department of Thoracic and Cardiovascular Surgery, Chonnam National University Hospital, Chonnam National University School of Medicine, 42 Jebong-ro, 501-757 Dong-gu, Gwangju South Korea; 2 Department of Thoracic and Cardiovascular Surgery, Seoul National University Bundang Hospital, Seongnam, South Korea; 3 Medical Research Collaborating Center, Seoul National University College of Medicine / Seoul National University Hospital, Seoul, South Korea; 4 Department of Thoracic and Cardiovascular Surgery, Chonnam National University Hwasun Hospital, Chonnam National University School of Medicine, Hwasun, South Korea

**Keywords:** Prolonged air leak, Lobectomy, Air leak grade

## Abstract

**Background:**

Results of studies to predict prolonged air leak (PAL; air leak longer than 5 days) after pulmonary lobectomy have been inconsistent and are of limited use. We developed a new scale representing the amount of early postoperative air leak and determined its correlation with air leak duration and its potential as a predictor of PAL.

**Methods:**

We grade postoperative air leak using a 5-grade scale. All 779 lobectomies from January 2005 to December 2009 with available medical records were reviewed retrospectively. We devised six ‘SUM’ variables using air leak grades in the initial 72 h postoperatively.

**Results:**

Excluding unrecorded cases and postoperative broncho-pleural fistulas, there were 720 lobectomies. PAL occurred in 135 cases (18.8%). Correlation analyses showed each SUM variable highly correlated with air leak duration, and the SUM_4to9_, which was the sum of six consecutive values of air leak grades for every 8 h record on postoperative days 2 and 3, was proved to be the most powerful predictor of PAL; PAL could be predicted with 75.7% and 77.7% positive and negative predictive value, respectively, when SUM_4to9_ ≥ 16. When 4 predictors derived from multivariable logistic regression of perioperative variables were combined with SUM_4to9_, there was no significant increase in predictability compared with SUM_4to9_ alone.

**Conclusions:**

This simple new method to predict PAL using SUM_4to9_ showed that the amount of early postoperative air leak is the most powerful predictor of PAL, therefore, grading air leak after pulmonary lobectomy is a useful method to predict PAL.

## Background

There is no clear consensus on the duration of prolonged air leak (PAL) [1], which is usually considered as lasting longer than 5 or 7 days postoperatively. Air leaking after pulmonary resection is natural, but its prolongation increases the risks of other pulmonary complications such as empyema [2] and unnecessarily lengthens hospital stay [3, 4]. Therefore, accurate prediction of PAL could enable early, selective postoperative management to prevent PAL possible, which in turn help to reduce the complication risks and hospital costs.

Many studies to elucidate the risk factors of PAL have been made to predict its occurrence [5–10], but the results were inconsistent and therefore, of limited use clinically. Thus, rather than identifying the risk factors, we sought to determine whether observing the pattern of postoperative air leak might be a more direct and accurate way, based on a simple assumption: ‘the larger, the longer’.

In this study, the authors developed a new quantitative scale to express the amount of early postoperative air leak to determine its correlation with air leak duration and possibility as a predictor of PAL.

## Methods

The medical records of 779 lobectomies conducted consecutively at our institution from January 2005 to December 2009 were reviewed retrospectively. This study was approved by Seoul National University Bundang Hospital’s Institutional Review Board, and the need for informed consent was waived (IRB number: B-1010-113-103).

### Grading of air leak and definition of time period ‘P’

**Fig. 1:**
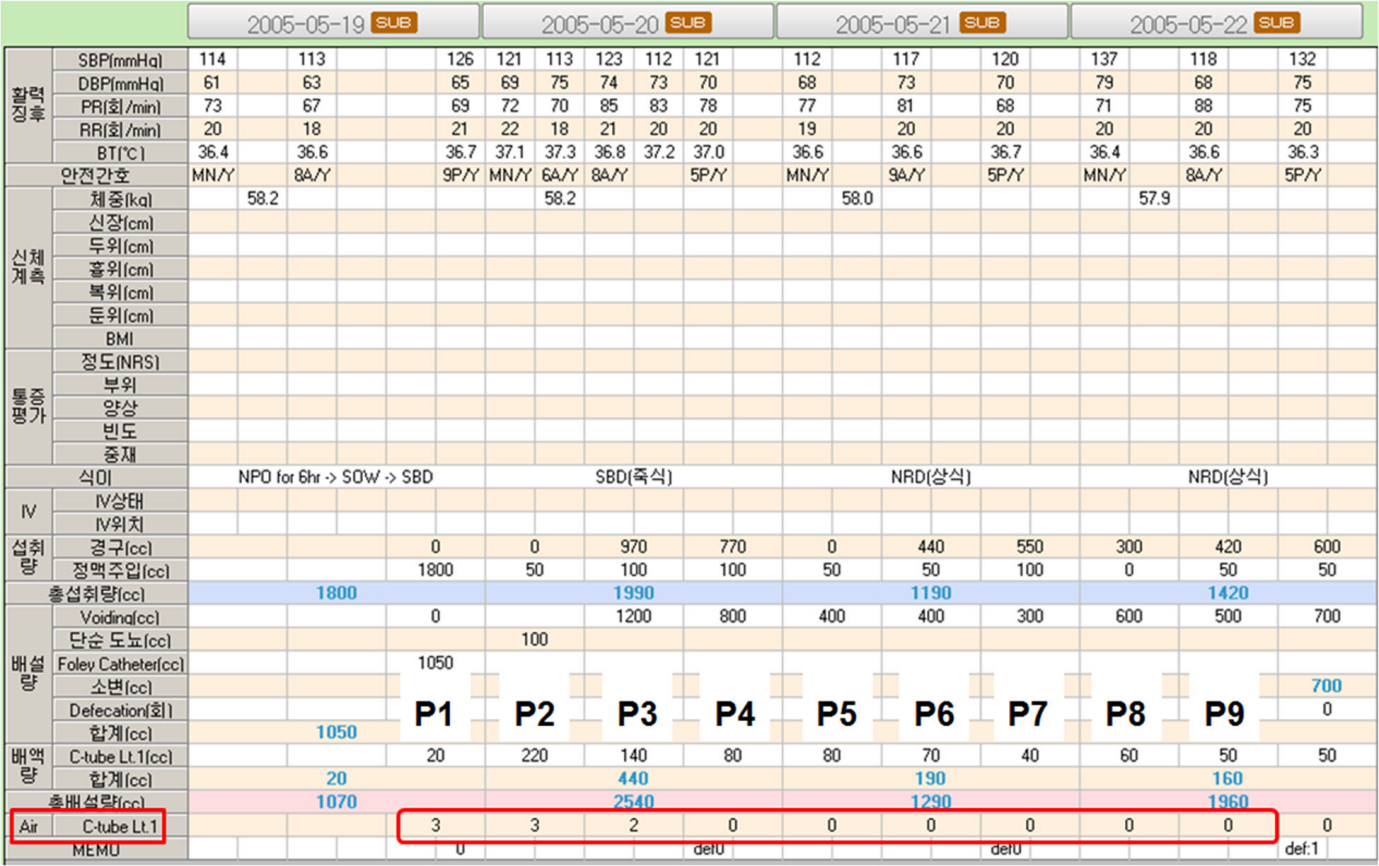
Actual air leak grading chart in our hospital. Air leak grades were recorded every 8 h (each “P”) postoperatively. In this case, air leak ceased at P6 and the SUM_4to9_ was 0

We began implementing a chest tube management protocol including quantitative assessment of air leak using a 5-grade scale (Table 1) and were able to evaluate almost all postoperative air leaks since December 2004. After pulmonary resection, in our hospital, air leak grades are recorded on the vital sheet along with blood pressure or heart rate. Air leak grading, based on evaluation of volitional coughing by the patient, was performed by specially trained general nurses and recorded every 8 h, because of their 3 shifts per day. Therefore, to facilitate postoperative data collection, the authors defined one 8-h period corresponding to one nursing shift as a time scale one ‘P’ and expressed every postoperative 8-h period as P1, P2, P3, etc. (Fig. 1).

**Table 1:** Air leak grading

Grade	Definition
0	No air bubble on three serial volitional coughs
1	More than one air bubbles on three serial volitional coughs
2	Persistent air bubbles on volitional coughs
3	Persistent, small amount of air bubbles on spontaneous respiration
4	Persistent, large amount of air bubbles on spontaneous respiration

### Definition of air leak cessation and PAL

We generally do not remove the chest tube until the drainage amount decreases to less than 150 – 200 mL within the previous 24 h. Therefore, chest tube removal time cannot reflect the time when air leak stops, so prior to performing this study, air leak cessation was necessary to be defined clearly based on the grading records. Air leak cessation was defined as air leak grade 0 or 1 being continued for 3 Ps (24 h). In cases with 2 chest tubes, air leak cessation was timed based on the late-ceased one. In this study, PAL was defined as air leak persisting more than 5 days (15 Ps).

### ‘SUM’ variables as predictors of PAL

For quantitative comparison of early postoperative air leak, we devised 6 ‘SUM’ variables, based on which we evaluated the degree of air leak from P1 to P9 (Table 2). In cases having 2 chest tubes, the larger one among the two values of air leak grade in the same P was selected for evaluation. Six SUM variables representing the amount of early postoperative air leak were examined respectively to see whether they correlated with air leak duration or not. Then, among the variables proved to correlate with air leak duration, the most optimal variable and its cutoff value for prediction of PAL were obtained. Other predictors for PAL derived from preoperative and intraoperative variables of this study cohort were also tested as to whether they had any additive effect on optimizing prediction of PAL.

**Table 2:** Definitions of 6 ‘SUM’ variables

Postoperative period	P1	P2	P3	P4	P5	P6	P7	P8	P9
Air leak grade^a^	0-4 (N1)	0-4 (N2)	0-4 (N3)	0-4 (N4)	0-4 (N5)	0-4 (N6)	0-4 (N7)	0-4 (N8)	0-4 (N9)
Variable	Definition								
SUM_1to3_	N1 + N2 + N3								
SUM_4to6_	N4 + N5 + N6								
SUM_1to6_	N1 + N2 + N3 + N4 + N5 + N6								
SUM_7to9_	N7 + N8 + N9								
SUM_4to9_	N4 + N5 + N6 + N7 + N8 + N9								
SUM_1to9_	N1 + N2 + N3 + N4 + N5 + N6 + N7 + N8 + N9								

^a^‘N’ represents the air leak grade, from 0 to 4, for the given postoperative period ‘P’

### Validation of the reliability of air leak grading

For 1 week while conducting this study, we had one or two pairs of nurses from each shift assess the air leak grade simultaneously but independently, and one of the authors (Jung Y) compared the two grades to monitor the agreement on air leak grading.

### Statistical analysis

Spearman rank correlation coefficients were calculated to investigate the correlation between each SUM variable and air leak duration. Receiver operating characteristic (ROC) curve analyses were performed and areas under the ROC curves (AUC) were compared to select the best predictive SUM variable, then its cutoff value that optimized both sensitivity and specificity was obtained.

The distribution of preoperative and intraoperative variables were compared between PAL (+) and PAL (−). Categorical variables were expressed as the frequency and percentage of cases and compared by either the χ^2^ test or Fisher’s exact test, as necessary. For a continuous variable with a nonnormalized distribution, data were reported as a median with range and compared by the Mann-Whitney U-test. The significant factors determined by a two-tailed nominal *P* value of <0.2 in univariable analysis were entered into a multivariable logistic regression to identify predictors of PAL. Stepwise logistic regression was performed to control multicollinearity among predictors and for each element remaining in the multivariable model, a *P* value, odds ratio (OR), and 95% confidence interval (CI) were calculated. The Hosmer-Lemeshow goodness-of-fit statistic was used to evaluate the model fit. The resultant predictors and the optimal SUM variable were combined to create a multivariable logistic regression, the AUC of which was compared with that of the optimal SUM variable regarding predictive ability [11]. A two-sided P value of <0.05 was considered to indicate a statistically significant difference. In order to assess the agreement on air leak grading, the weighted kappa statistic was performed. The analysis was carried out using PASW Statistics, version 17.0 (SPSS Inc.; Chicago, IL) and SAS, version 9.1 (SAS Institute; Cary, NC).

## Results

Excluding the cases of 53 lacking graded records, 3 having 3 chest tubes, 2 postoperative broncho-pleural fistulas, and 1 followed by sequential operation in a situation where air leak after initial lobectomy had not ceased, a total of 720 consecutive lobectomies were included in this study. The most common preoperative diagnosis was malignant lung neoplasm (including pulmonary metastasis) occupying 89.9%. The number of cases with single chest tube was 372 (51.7%). Other characteristics of this study cohort are summarized in Table 3.

**Table 3:** Baseline characteristics and results of univariable analyses

Variables	No. of cases (%)			*P* value^a^
	All	PAL (−)	PAL (+)	
	(*N* = 720)	(*N* = 585)	(*N* = 135)	
Age^b^ (years)	64 (7–85)	64 (7–85)	66 (14–82)	<0.001^c^
Male	464 (64.4%)	354 (60.5%)	110 (81.5%)	<0.001
Preoperative medication				
Aspirin or NSAIDs	98 (13.6%)	80 (13.7%)	18 (13.3%)	0.92
Steroid (including inhalator)	15 (2.1%)	9 (1.5%)	6 (4.4%)	0.05^d^
Neoadjuvant chemotherapy	50 (6.9%)	39 (6.7%)	11 (8.1%)	0.54
Preoperative spirometry				
FEV_1_/FVC < 70%	259 (36.0%)	201 (34.4%)	58 (43.0%)	0.04
FVC% < 80	76 (10.6%)	62 (10.6%)	14 (10.4%)	0.90
FEV_1_% < 80	108 (15.0%)	81 (13.8%)	27 (20.0%)	0.04
D_L_CO% < 80	103 (14.3%)	81 (13.8%)	22 (16.3%)	0.34
Underlying DM	101 (14.0%)	83 (14.2%)	18 (13.3%)	0.80
Preoperative Hb < 10 g/dL	29 (4.0%)	23 (3.9%)	6 (4.4%)	0.41^d^
Preoperative albumin < 3.0 g/dL	12 (1.7%)	10 (1.7%)	2 (1.5%)	0.32^d^
BMI < 25.5 kg/m^2^	523 (72.6%)	416 (71.1%)	107 (79.3%)	0.07
Open thoracotomy	375 (52.1%)	299 (51.1%)	76 (56.3%)	0.28
Right side operation	456 (63.3%)	352 (60.2%)	104 (77.0%)	<0.001
Upper lobectomy	372 (51.7%)	293 (50.1%)	79 (58.5%)	0.08
Combined chest wall resection	21 (2.9%)	17 (2.9%)	4 (3.0%)	1.00^d^
Pleural adhesion (+)	338 (46.9%)	256 (43.8%)	82 (60.7%)	<0.001
Incomplete fissure (+)	362 (50.3%)	284 (48.5%)	78 (57.8%)	0.05

*NSAID* denotes non-steroidal anti-inflammatory drug, *DM* diabetes mellitus, *Hb* hemoglobin, *BMI* body mass index. In spirometry variables, *FEV*_*1*_ denotes forced expiratory volume in one second, *FVC* forced vital capacity, *D*_*L*_ *CO* diffusing capacity of the lung for carbon monoxide. Suffix ‘%’ means ‘% of predicted normal’

^a^ *P* values are for comparison between PAL (−) and PAL (+)

^b^Exceptionally, ages are expressed as ‘median (range)’

^c^Mann-Whitney U-test was performed

^d^Fisher’s exact test was performed. In other cases, χ^2^ test was performed

Median duration of postoperative air leak was 2.7 (0.7–58.0) days and the median of chest tube removal was at postoperative day (POD) 4.7 (0.7–67.0), and that of discharge was POD 7 (2–187). The numbers of cases with persistent air leak at P4, P7, P10, P13 and P16 are 577, 410, 275, 184, and 127, respectively. A total of 127 cases with persistent air leak at P16 were considered as PAL and 8 other cases were also regarded as PAL despite their air leak duration being ≤ 15 Ps, because pleurodesis had been conducted for prophylaxis of PAL, so the total occurrence of PAL in this study cohort was 135 (18.8%).

### The larger, the longer?

Based on 526 cases having a chest tube beyond POD 3, correlation analyses showed that each SUM variable strongly correlated with air leak duration. The Spearman rank correlation coefficient ranged from the lowest of 0.71 for SUM_1to3_ to the highest of 0.87 for SUM_4to9_ (Table 4), which is thought to not only mean that all SUM variables can play a role as predictors of PAL, but also support the basic assumption of this study that the larger the early postoperative air leak, the longer it will persist.

**Table 4:** Statistical values of SUM variables

Variables	Spearman rank correlation coefficient	Calculated AUC
SUM_1to3_	0.71	0.71
SUM_4to6_	0.81	0.78
SUM_7to9_	0.83	0.81
SUM_1to6_	0.81	0.77
**SUM**_**4to9**_	**0.87**	**0.82**
SUM_1to9_	0.87	0.81

*AUC* denotes area under the ROC curve

The bold data show that SUM4to9 is the most strongly associated parameter with the air leak duration (i.e. the highest Spearman correlation coefficient) and the most powerful predictor of PAL (i.e. the largest AUC)

### Prediction of PAL by the SUM scale

PAL occurred in 134 cases (48.7%) out of 275 cases with persistent air leak at P10. ROC curves were generated for each SUM variable. The calculated AUC revealed that SUM_4to9_ to be the most powerful predictor of PAL (the largest AUC; 0.819. Table 4), and PAL could be predicted with 76.9% sensitivity and 76.6% specificity with 75.7% positive predictive value and 77.7% negative predictive value, when SUM_4to9_ ≥ 16.

**Fig. 2:**
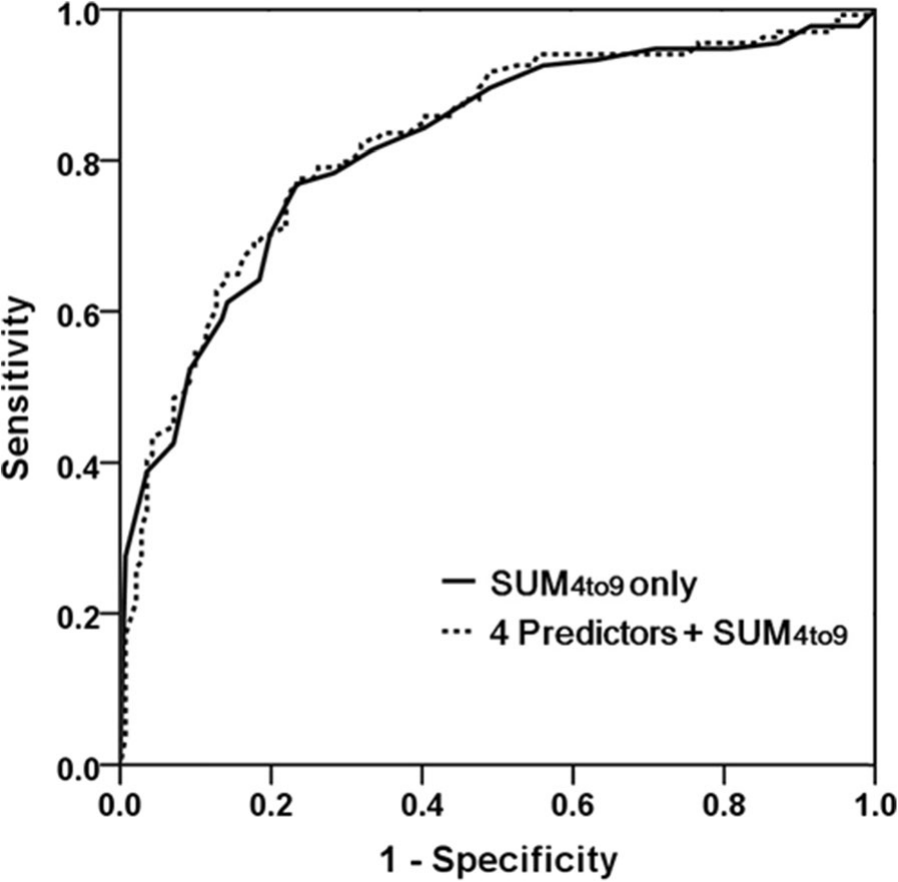
Comparison of the 2 AUCs. The solid line represents the ROC curve derived from SUM_4to9_ only (AUC 0.82) and the dotted line, combination of 4 perioperative risk factors of PAL and SUM_4to9_ (AUC 0.83). There was no statistically significant difference (*P* = 0.46)

Univariable analysis revealed significant differences between PAL (+) and PAL (−) in regard to age, male gender, preoperative steroid medication, FEV_1_/FVC < 70%, FEV_1_% < 80, BMI < 25.5 kg/m^2^, right side operation, upper lobectomy, presence of pleural adhesion, and incomplete fissure (Table 3). Stepwise logistic regression analysis with each associated variable determined by univariable analysis revealed only male gender, right side operation, presence of pleural adhesion, and BMI < 25.5 kg/m^2^ as significantly associated with PAL (Table 5). When these 4 predictors were combined with SUM_4to9_ into a multivariable logistic regression model to predict PAL, there was no significant increase in predictability compared with that of SUM_4to9_ alone (calculated AUC 0.83 vs. 0.82 respectively, *P* = 0.46) (Fig. 2).

**Table 5:** Results of multivariable logistic regression

Predictors	OR	*P* value	95% CI	
			Lower	Upper
Right side operation	2.37	<0.001	1.52	3.70
Male	2.66	<0.001	1.65	4.28
Pleural adhesion	1.76	0.005	1.18	2.62
BMI < 25.5 kg/m^2^	1.72	0.02	1.08	2.75

*OR* denotes odds ratio, *CI* confidence interval, *BMI* body mass index

### Reliability of air leak grading

A total of 33 pairs of air leak grades were collected. Twenty two pairs of ward nurses participated in the grading. There were 7 disagreements on air leak grading, but the degrees of difference were never more than a single grade. The weighted kappa coefficient between the 2 measurements of air leak grade was 0.88 (95% CI: 0.79–0.96), indicating a very good interobserver agreement [12].

## Discussion

We set out to determine whether our newly developed scale could be useful as a predictor of PAL. Although there have been many studies to predict PAL, most of which have tried to determine predictive risk factors by comparing PAL groups with other groups and reported various risk factors such as poor pulmonary function [6, 8–10], poor nutrition [7], or specific operative findings [8, 10], but their results were not consistent with each other and therefore are of limited clinical use. Brunelli et al. [10] in 2010, unlike previous studies, reported a risk factor scoring system for prediction of PAL. He elicited 4 predictors from a study group of 658 lobectomy patients: age > 65 years, presence of pleural adhesion, FEV_1_% < 80, and BMI < 25.5 kg/m^2^, and different scores were given to the factors according to their weights. The scoring system was validated externally in 233 other hospital patients. However it also had a limitation because of its low positive predictive value of about 25%.

Various investigators have examined the concept that grading the amount of early postoperative air leak might be helpful in predicting PAL [13, 14]. In 2001, Cerfolio et al. [13] reported that PAL can be predicted by air leak grade on POD 1. In their study, a commercially available air-leak meter, scoring leaks from 1 to 7 with 7 being the highest, was used. However, originally his work aimed to evaluate the effectiveness of water seal for stopping air leaks. Thus he did not give much weight to air leak grading as a predictor of PAL. In addition, the air leak grading system devised by Cerfolio et al. requires special equipment, and thus the system is not widely used.

To the best of our knowledge, the present study is the first practical attempt to focus only on quantifying air leakage in the early postoperative period to predict PAL. The easy-to-use variable, SUM_4to9_, has the highest positive predictive value among reports until now. Our air leak grading system, to obtain SUM_4to9_, needs no special equipment, and yet it is very convenient to apply in the clinical field. Based on our results, we can now decide whether to wait or perform a reintervention (e.g. pleurodesis or redo surgery) for air leak cessation on POD 3.

In addition to proposing a practical and effective method to predict PAL, this study tangibly confirms our hypothesis that the amount of early postoperative air leak predicts air leak duration, by correlation analyses of SUM variables with air leak duration. Furthermore, it reveals that other preoperative or intraoperative variables do not increase the predictive power of SUM_4to9_. These findings can be integrated to mean that 1) grading postoperative air leak might be the only factor needed to predict PAL, and 2) the effects of various possible factors contributing to prolongation of air leak might combine to result in the grade of early postoperative air leak. Therefore, future studies aiming at providing more accurate prediction of PAL will have to focus on the evaluation of early postoperative air leak in terms of when, by what method, and how frequently it should be measured, not on other indirect factors.

However, this study has the following potential limitations. First of all, the retrospective nature of the study might have incurred some problems in defining and recording the variables. In particular, since air leak cessation was determined retrospectively from medical records, there might have been some discrepancy between the real and the defined cessations. Essentially, our air leak grading is based on subjective assessment, albeit by specially trained nurses, so some might question its reliability. However, over the past several years, we have empirically recognized that its simplicity enables strong interobserver agreement, as shown in the test to see the reliability of our air leak grade. Recently developed digital airflowmetry may be helpful in further increasing interobserver agreement in future studies.

## Conclusions

We developed an easy-to-use method to predict PAL using a new scale, SUM_4to9_, derived from our own air leak grading protocol. This study proved that the amount of early postoperative air leak is the most powerful predictor of PAL, therefore, grading air leak after pulmonary lobectomy is a useful method to predict PAL.

## Abbreviations

AUC: Area under the ROC curve
BMI: Body mass index
D_L_CO%: % of predicted normal diffusing capacity of the lung for carbon monoxide
DM: Diabetes mellitus
FEV_1_%: % of predicted normal forced expiratory volume in one second
FVC%: % of predicted normal forced vital capacity
Hb: Hemoglobin
NSAID: Non-steroidal anti-inflammatory drug
PAL: Prolonged air leak
POD: Postoperative day
ROC curve: Receiver operating characteristic curve
VATS: Video-assisted thoracoscopic surgery
